# Cinchonida and Cinco-quinine

**Published:** 1877-10-20

**Authors:** 


CINCHONIDIA AND CINQUO-QUININE.—The discussion touching the relative merits of the two preparations above named, still goes on, as may be seen, by an interesting paper in our present issue. If the high price of the Sulphate of Quinine shall have the effect of leading to experimental tests, and of enlarging our knowledge of the peculiar and varied properties of the Cinchona bark and its alkaloids, it will, after all, prove a blessing to the profession and to the country.

